# Early versus delayed oral sialagogues for the prevention of salivary gland dysfunction after radioiodine therapy in differentiated thyroid cancer: a systematic review

**DOI:** 10.1007/s12020-026-04727-z

**Published:** 2026-08-03

**Authors:** Andressa Frankowski Dagostin, Novel Yourdhan Rehandhika, Ana Luiza Potiguara de Souza, Soemiwati Holland

**Affiliations:** 1 San Raffaele University, Milan, Italy; 2 Department of Nuclear Medicine and Molecular Theranostics, Faculty of Medicine, Padjadjaran University, Dr. Hasan Sadikin General Hospital, Bandung, West Java, Indonesia; 3 Catholic University of Brasilia (UCB), Brasilia, Brazil; 4 Hackensack Meridian Health Jersey Shore University Medical Center, Neptune, NJ United States of America; 5 Hackensack Meridian School of Medicine, Clifton, NJ United States of America

**Keywords:** Salivary gland dysfunction, Radioiodine therapy, Sialagogues, Sialadenitis, Thyroid cancer

## Abstract

**Background:**

Salivary gland dysfunction is a recognized complication of radioactive iodine (RAI) therapy for differentiated thyroid cancer. Oral sialagogues are commonly recommended, but their optimal timing remains uncertain. This systematic review compares early (<24 h) versus delayed (>24 h) sialagogue initiation.

**Methods:**

A literature search was conducted in Embase, PubMed, CINAHL, and Cochrane from inception to January 2026. “Early” protocols initiated stimulation within 24 h post-RAI, while “delayed” protocols initiated after 24 h. Methodological quality and risk of bias were assessed using Cochrane’s ROBINS-I (Risk Of Bias In Non-randomized Studies of Interventions, 2016) tool.

**Results:**

Three observational studies were included, comprising 818 adults with reported mean ages of 43–59 years. Interventions consisted primarily of lemon candy or oral vitamin C tablets. Primary outcomes included acute sialadenitis (pain and/or swelling), taste dysfunction, and xerostomia. Findings regarding acute salivary gland outcomes were inconsistent. Two earlier studies reported higher rates of acute sialadenitis with early stimulation (54.7–63.8% vs. 34.4–36.8%), taste dysfunction (37.5–39.0% vs. 18.8–25.6%), and xerostomia (23.8–46.9% vs. 11.2–29.7%) compared with delayed initiation. In contrast, the most recent propensity score-matched study, which evaluated a composite endpoint of acute salivary gland damage, reported lower event rates with early stimulation (4.78% vs. 15.22%).

**Conclusion:**

This review underscores how evidence regarding the optimal timing of sialagogue initiation after RAI remains inconsistent. These heterogeneous findings highlight the need for high-quality randomized controlled trials to establish evidence-based recommendations for sialagogue timing after RAI therapy.

**Supplementary Information:**

The online version contains supplementary material available at 10.1007/s12020-026-04727-z.

## Introduction

Differentiated thyroid cancer (DTC), subdivided into papillary and follicular thyroid carcinomas, represents the most common endocrine malignancy worldwide [1]. The global incidence of DTC has risen steadily over recent decades, driven in part by increased awareness and detection of small, subclinical tumors through widespread use of high-resolution imaging [2, 3]. Despite this rising incidence, DTC is generally associated with an excellent prognosis, with 10-year disease-specific survival exceeding 90% when appropriately treated [4, 5]. Standard management includes surgical resection, often followed by radioactive iodine therapy (RAI) in selected patients to ablate residual thyroid tissue and reduce the risk of recurrence [6].

Unlike in undifferentiated thyroid cancer, DTC cells retain expression of the sodium–iodide symporter (NIS), enabling selective uptake of RAI and facilitating targeted cytotoxicity [7]. However, NIS is also physiologically expressed in salivary glands, leading to unintended iodine accumulation and radiation-induced glandular injury [8]. Hence, salivary gland toxicity is a common adverse effect of RAI therapy [9]. It can induce chronic salivary dysfunction, clinically manifesting as sialadenitis, taste dysfunction, and xerostomia, all of which may persist long term and significantly impair patients’ quality of life [10, 11]. The severity of damage appears to correlate with cumulative RAI dose and iodine retention time within glandular tissue [11, 12].

To mitigate these adverse effects on salivary gland function, oral sialagogues, most commonly sour candy, lemon juice, or vitamin C, are routinely recommended in clinical practice [13]. The rationale for their use is to increase salivary flow, thereby accelerating RAI clearance from the glands and reducing radiation exposure [14]. However, stimulation may also transiently increase salivary gland perfusion and potentially enhance iodine uptake during periods of peak systemic radioactivity [14]. As a result, the optimal timing of sialagogue initiation remains controversial, with clinical practice varying widely between institutions. This systematic review aims to evaluate whether the timing of sialagogue initiation (early vs. delayed) influences the incidence and severity of salivary gland dysfunction following RAI therapy for DTC.

## Methods

This systematic review was prospectively registered in the International Prospective Register of Systematic Reviews (PROSPERO) under protocol number CRD420261293253. This study was designed in accordance with the Cochrane Handbook for Systematic Reviews of Interventions and adheres to the Preferred Reporting Items for Systematic Reviews and Meta-Analyses (PRISMA) guidelines [15]. A PRISMA checklist was completed and can be found in the Supplemental Appendix A.

### Literature search strategy

A comprehensive literature search was performed in the electronic databases CINAHL, EMBASE, PUBMED, and Cochrane Library from inception up to January 2026. The electronic search was supplemented with an examination of the reference lists of relevant articles and reviews, and was restricted to peer-reviewed journal articles or articles in press. The complete search strategy is available in Supplemental Appendix B.

### Eligibility criteria

Eligible studies were randomized controlled trials (RCTs) or observation cohorts involving adult patients that had undergone RAI therapy with I-131 after thyroidectomy for DTC and were given oral sialagogues either within or after 24 h of I-131 administration. Eligible sialagogues included, but are not limited to, lemon candy, lemon juice, ascorbic acid, citrus fruits, or chewing gum. Studies with patients with a history of salivary gland disorders, previous head and neck external beam radiation, or those taking medications known to significantly alter salivary flow were excluded.

Studies were excluded if they reported only single-arm data without a comparator or were case reports, case series, narrative or systematic reviews, trial protocols, letters to the editor, editorials, abstracts, or duplicate publications. No restrictions were applied for language, publication status, sample size, or follow-up duration. Justifications for excluded full-text articles are provided in the Supplemental Appendix C (Supplemental Table C.1).

### Study selection

The articles identified were imported into Rayyan software for article selection, which was independently performed by two authors (A.F.D. and N.Y.R.) using the predefined eligibility criteria. After removing duplicates, titles and abstracts were screened. If the title and abstract were insufficient to make a decision, the full text was assessed for inclusion. Disagreements were resolved by consensus. Data extraction was conducted independently by two authors (N.Y.R. and S.H.), including study characteristics, baseline demographics, operative details, and all reported outcomes of interest. The salivary gland dysfunction outcomes were acute sialadenitis (pain/swelling*)*, taste dysfunction, and xerostomia. Outcome definitions for each study are summarized in the Supplemental Appendix D.

### Risk of bias and quality assessment

To evaluate the methodological quality of the included studies, the Cochrane Risk Of Bias In Non-randomized Studies of Interventions (ROBINS-I, 2016) tool was used, which rates studies as having low, moderate, serious, or critical risk of bias [16]. This tool assesses potential bias across several domains, including confounding, selection of participants, classification of interventions, deviations from intended interventions, missing data, outcome measurement, and selective reporting of results. Graphical summaries of the risk-of-bias assessments were produced using the robvis (Risk-of-Bias VISualization) tool [17]. Three authors (A.F.D., N.Y.R., and S.H.) independently performed the risk-of-bias assessments, and discrepancies were resolved through consensus.

## Results

**Fig. 1:**
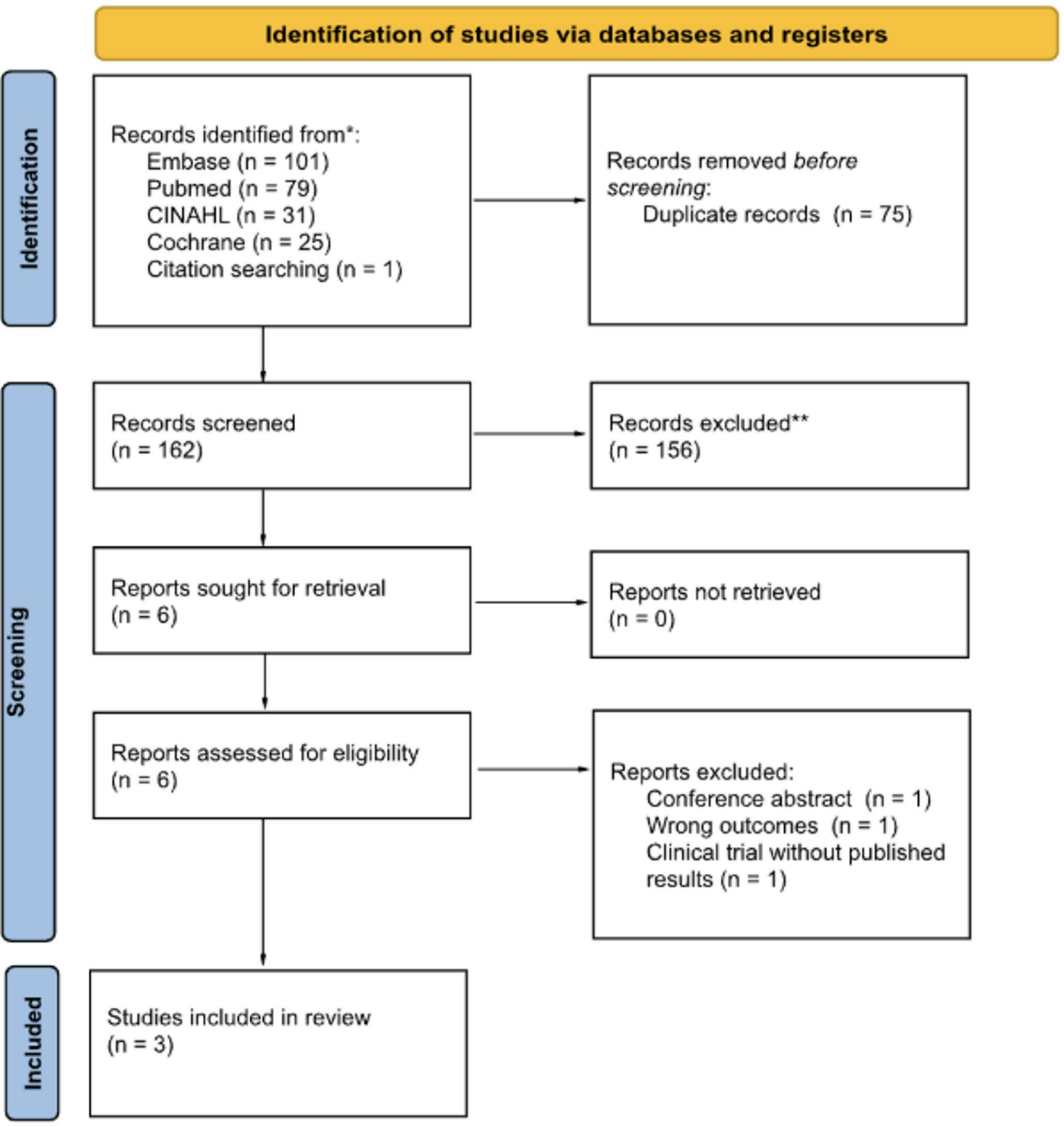
PRISMA 2020 flow diagram of included studies

A total of 236 studies were identified in the initial literature search, with 230 excluded after duplicate removal and screening based on title and abstract. Six studies underwent full-text review, three of which met the inclusion criteria and were included in this review [14, 18, 19]. The PRISMA flowchart is depicted in Fig. 1. Full texts of all eligible articles were retrieved through institutional access, and no study was included based on abstract-only data.

### Characteristics of included studies

We included three observational studies published between 2005 and 2022, with a combined population of 818 patients, of whom 402 (49.75%) belonged to the “early” sialagogue administration group [14, 18, 19]. Mean age, where reported, ranged from 43 to 58.5 years and participants were predominantly female, with female proportions ranging from approximately 71% to 89%.

The exact timing protocols differed between included studies, since Nakada et al. [14] and Reza et al. [19] initiated early stimulation at 1 h and delayed stimulation at 24 h, whereas Liu et al. [18] initiated early stimulation at 2 h and delayed stimulation at 24 h. Vitamin C was used by Liu et al. [18] and Reza et al. [19], while Nakada et al. [14] used lemon candy. Stimulation intensity also varied, including vitamin C 250 mg every 3 h during the daytime for 7 days in Reza et al. [19]; one to two lemon candies every 2–3 h during waking hours for 5 days in Nakada et al. [14], and vitamin C 100 mg hourly while awake for 5 days in Liu et al. [18]. Administered RAI activity was similar between groups in Nakada et al. (3.96 ± 0.41 vs. 3.87 ± 0.65 GBq) and balanced after propensity score matching in Liu et al. (110 [50] vs. 100 [50] mCi; approximately 4.07 [1.85] vs. 3.70 [1.85] GBq), whereas RAI activity was not reported by Reza et al. Baseline patient characteristics and detailed stimulation protocols are summarized in Table 1.

**Table 1:** Characteristics of included studies

Author; Country; Publication Year	Study Design	Timing Definition	Sample Size (*n*) Early/Delayed	Age (mean) Early/Delayed	Gender (% Males) Early/Delayed	RAI Dose Early/Delayed (GBq)	Stimulation Agent
Reza et al.; Bangladesh; 2018	Prospective quasi-experimental study	Early: < 1 h Delayed: > 24 h	64/64	43/43^b^	NA	NA	Vitamin C
Nakada et al.; Japan; 2005	Prospective cohort	Early: < 1 h Delayed: > 24 h	105/125	55.2/58.5	14.3/11.2	3.96/3.87	Lemon Candy
Liu et al.; China; 2022	Retrospective cohort	Early: < 2 h Delayed: > 24 h	230/230	45.06/45.29	28.7/29.1	4.07/3.70^b^	Vitamin C

h: hours; NA: not available

^a^Mean age estimated from the reported age range using midpoint approximation

^b^RAI activities originally reported in millicuries (mCi) and were converted to gigabecquerels (GBq) using 1 mCi = 0.037 GBq

### Quality assessment

Overall, all studies were judged to have moderate risk of bias. Confounding was the primary source of bias, reflecting their non-randomized design. Additional concerns were due to deviations from intended interventions, rated as moderate in the two earlier studies but low in the most recent study. Bias related to selection of participants, classification of interventions, and missing data was consistently rated as low (Supplemental Appendix E, Supplemental Figure E.1).

### Acute sialadenitis or acute salivary gland damage

Outcome definitions differed across studies. Reza et al. [19] and Nakada et al. [14] reported clinically defined sialadenitis, primarily based on salivary gland pain and/or swelling. Reza et al. observed sialadenitis in 54.7% of patients in the early group versus 34.4% in the delayed group (*p* = 0.021) [19]. Similarly, Nakada et al. found that 63.8% of the early group developed sialadenitis compared with 36.8% in the delayed group (*p* < 0.001) [14].

Conversely, Liu et al. evaluated acute salivary gland damage rather than isolated clinically diagnosed sialadenitis. In that study, salivary gland damage required both compatible clinical symptoms and objective dynamic salivary gland scintigraphy dysfunction. Acute salivary gland damage was observed in 4.78% of the 2-hour group compared with 15.22% of the 24-hour group (*p* < 0.001) [18].

### Taste dysfunction

Two studies reported taste dysfunction as a significant adverse effect more prevalent in the early stimulation group. Reza et al. noted taste dysfunction in 37.5% of the early group versus 18.8% of the delayed group (*p* = 0.018) [19]. Similarly, Nakada et al. found a higher incidence in the early group (39.0%) compared to the delayed group (25.6%), with a statistical significance of *p* < 0.01 [14].

### Xerostomia

Xerostomia was reported by two studies, with findings favoring delayed initiation. Nakada et al. reported that dry mouth was more frequent in the early group (23.8%) than in the delayed group (11.2%) (*p* < 0.005) [14]. Reza et al. found that dry mouth at 3 months was higher in the early group (46.9%) compared to the delayed group (29.7%) (*p* = 0.045) [19].

### Gland-specific findings

Gland-specific data were limited and were not consistently reported across studies. Nakada et al. reported parotid and submandibular gland involvement on scintigraphy, with bilateral parotid involvement most frequently observed, followed by bilateral submandibular involvement [14]. In contrast, Reza et al. did not provide separate quantitative parotid versus submandibular analyses, although obstructive parotitis was reported at 6 months [19]. Liu et al. provided separate scintigraphic assessments for the parotid and submandibular glands. Functional impairment rates did not significantly differ between the 2-hour and 24-hour groups for either gland, but submandibular concentration indices decreased significantly after RAI in both timing groups [18]. Formal synthesis by gland type was not possible since gland-specific outcomes were reported inconsistently.

## Discussion

This systematic review evaluated the timing of oral sialagogue initiation following post-thyroidectomy RAI therapy and identified a limited and heterogeneous body of evidence addressing this clinically relevant question. Notably, only three studies met inclusion criteria, highlighting a substantial and persistent knowledge gap regarding the optimal timing of salivary stimulation after RAI administration.

Salivary gland dysfunction is a well-documented complication of RAI therapy, although reported incidence varies widely across studies due to heterogeneity in outcome definitions, diagnostic methods, RAI dosing, and follow-up duration. Previous literature has reported rates of sialadenitis ranging from 2% to as high as 67% across different studies [9, 20–22]. Studies have also shown that the risk of sialadenitis is dose-dependent, with higher cumulative RAI doses associated with increased incidence and severity of glandular damage [23, 24].

A key finding of this review was an inconsistency in outcomes related to acute sialadenitis. Two earlier studies reported higher rates of sialadenitis with early sialagogue administration, whereas the most recent study reported significantly lower incidence with earlier intervention. Several factors may contribute to these divergent findings. First, the studies differed in methodological approach and study design. The most recent study applied propensity score matching to adjust for baseline characteristics, whereas earlier studies relied on unadjusted comparisons, increasing the potential for confounding. Second, differences in patient populations may have influenced susceptibility to salivary gland injury. Factors such as age, sex distribution, baseline salivary gland function, hydration status, and prior comorbidities have been associated with variability in salivary gland complications following RAI therapy in previous literature [23, 24]. Third, as previously mentioned, variations in administered RAI activity may have contributed to outcome differences, as the risk of salivary gland toxicity has been shown to increase with higher cumulative radioiodine doses [23, 24]. Differences in stimulation protocols may also have played a role, including the type of sialagogue used (e.g., vitamin C versus lemon candy), the exact timing of initiation, and the duration or frequency of stimulation. Finally, heterogeneity in outcome definitions and assessment methods may have affected the reported incidence of sialadenitis. While some studies relied primarily on patient-reported symptoms, others incorporated objective imaging-based assessments of salivary gland function, which may capture different aspects of glandular injury. Collectively, these methodological and clinical differences likely contributed to the heterogeneity observed across studies.

Two earlier studies reported higher rates of sialadenitis with early sialagogue administration, whereas the most recent study reported lower incidence of composite acute salivary gland damage with initiation at 2 h [14, 19, 18]. Several factors may contribute to these divergent findings. First, the studies differed in methodological approach. Liu et al. used propensity score matching for age, sex, TSH level, number of surgeries, Hashimoto thyroiditis, clinical stage, metastatic status, and administered RAI dose [18], whereas Reza et al. and Nakada et al. relied on unadjusted comparisons [14, 19]. Balancing these variables may reduce confounding related to disease severity and treatment intensity. However, residual confounding from unmeasured variables remains possible. Second, the outcome definitions differed: Reza et al. and Nakada et al. primarily assessed clinical sialadenitis based on pain and/or swelling, while Liu et al. used a composite definition requiring both symptoms and scintigraphic dysfunction. Because this composite endpoint differs from the clinical sialadenitis definitions used by Reza et al. and Nakada et al., the findings reported by Liu et al. should be interpreted as related to, but not directly equivalent to, the outcomes assessed in the earlier studies. In Liu et al., acute salivary gland damage was defined as pain and swelling occurring within days to one month after RAI administration, whereas chronic damage (occurring after one month) encompassed decreased salivary secretion, taste changes, xerostomia, dental caries, halitosis, and salivary adenitis.

Among the studies reporting administered activity, RAI doses were broadly comparable and within the range typically used for thyroid remnant ablation. The study conducted by Nakada et al. reported 3.96 ± 0.41 versus 3.87 ± 0.65 GBq [14], and Liu et al. demonstrated approximately 4.07 versus 3.70 GBq after propensity score matching [18], both within the range typically used for thyroid remnant ablation. In contrast, the RAI activity was not reported by Reza et al. [19]. Therefore, residual confounding by administered activity cannot be excluded. Finally, differences in sialagogue type, dosing intensity, frequency, and duration may also have contributed to outcome variability across studies.

The proposed mechanism underlying oral sialogogue use is to stimulate salivary flow, thereby accelerating radioactive iodine clearance from the salivary glands and potentially reducing absorbed dose [25]. However, early stimulation may also increase gland perfusion and functional activity during periods of peak systemic radioactivity, potentially enhancing iodine delivery to glandular tissue [14]. This competing physiological effect may help explain why earlier observational studies reported worse outcomes with early stimulation [18, 19]. Taken together, these findings suggest that the balance between enhanced clearance and increased iodine uptake is likely time-dependent and may vary according to the precise timing of stimulation relative to RAI administration.

In contrast to the inconsistent findings observed for acute sialadenitis or acute salivary gland damage, outcomes such as taste dysfunction and dry mouth/xerostomia more consistently favored delayed stimulation in the two earlier studies [14, 19]. However, Liu et al. reported lower chronic salivary gland damage with the 2-hour protocol, again using a composite clinical and scintigraphic definition [18]. These findings suggest that symptom-based outcomes and imaging-defined salivary gland injury likely represent related but distinct clinical constructs and therefore should not be considered directly interchangeable. Differences in follow-up duration and assessment methods further limit direct comparison across studies. Gland-specific analyses were limited and inconsistently reported; where available, no significant difference between timing groups was observed for either parotid or submandibular functional impairment, although submandibular concentration indices decreased after RAI administration in both groups.

This review has several important limitations. First, only three nonrandomized comparative studies met inclusion criteria, and no RCTs were identified. Second, substantial clinical and methodological heterogeneity, including differences in study design, sialagogue type, RAI activity, timing definitions, stimulation intensity, outcome measurement, and follow-up duration, precluded quantitative synthesis. Third, confounding variables such as hydration status, baseline gland function, cumulative RAI exposure, and supportive measures were inconsistently reported and adjusted for. Fourth, outcome definitions varied across studies; in particular, clinically diagnosed sialadenitis in the two earlier studies was not identical to the composite salivary gland damage endpoint used by Liu et al. Fifth, gland-specific data for parotid versus submandibular glands were limited and inconsistently reported, preventing meaningful synthesis by gland type. Finally, because one study did not report administered RAI activity [19], dose-related salivary toxicity could not be fully evaluated across all cohorts.

Guidance from major professional bodies regarding the optimal timing of oral sialogogue initiation remains limited and inconsistent. The 2025 American Thyroid Association (ATA) guidelines (Recommendation 40) recommend counseling patients regarding the risk of salivary gland morbidity and implementing general preventive measures, such as adequate hydration [26]. However, the guidelines conclude that current evidence is insufficient to recommend for or against specific sialogogue modalities or timing strategies. They cite conflicting evidence, including one study suggesting that sour candy initiated within one hour of RAI may worsen salivary gland injury compared with delaying stimulation for 24 h, whereas others have reported transient reductions in parotid radiation exposure with early, repeated lemon juice stimulation [26]. Similarly, neither the European Association of Nuclear Medicine (EANM) practice guideline [27] nor the earlier EANM guideline for DTC [13] specifies a preferred timing protocol, and the 2020 American Head and Neck Society multidisciplinary consensus statement likewise does not make a recommendation regarding sialogogue timing [28]. Given this uncertainty, and consistent with the findings of the present review, an individualized approach that incorporates established preventive measures while acknowledging the absence of high-quality evidence regarding optimal sialogogue timing appears most appropriate until adequately powered RCTs become available.

In summary, due to the limited, heterogeneous, and partially conflicting evidence, this systematic review cannot provide a definitive recommendation on whether early or delayed sialagogue initiation is more beneficial following RAI therapy. The findings underscore the need for high-quality trials designed to determine whether sialagogue timing could be a factor associated with improved or worse salivary gland outcomes as current data remains limited. Future studies should aim to use standard ablative RAI doses and carefully stratify groups according to timing to facilitate comparison and synthesis of findings and enhance the reliability of outcomes.

## Conclusion

In summary, current evidence regarding the optimal timing of acidic stimulation after RAI therapy is limited and conflicting. While earlier studies suggest potential harm with immediate stimulation, more recent propensity-adjusted data suggest possible benefit. Given the scarce and observational nature of available evidence, no definitive recommendation can be made. Well-designed randomized controlled trials are needed to establish evidence-based guidelines for the optimal timing of sialagogue administration to mitigate salivary gland dysfunction post-RAI therapy.

## Supplementary Information

Below is the link to the electronic supplementary material.

Supplementary Material 1

Supplementary Material 2

## Abbreviations

RAI: radioactive iodine
DTC: differentiated thyroid cancer
RCT: Randomized Controlled Trial
ROBINS-I: Risk Of Bias In Non-randomized Studies of Interventions
NIS: sodium–iodide symporter
PRISMA: Preferred Reporting Items for Systematic Reviews and Meta-Analyses
ATA: American Thyroid Association
EANM: European Association of Nuclear Medicine
