# Supplementary material for: Early versus delayed oral sialagogues for the prevention of salivary gland dysfunction after radioiodine therapy in differentiated thyroid cancer: a systematic review
Source: Endocrine. 2026 Aug 3;91(1):253. doi: 10.1007/s12020-026-04727-z (PMC13433636; doi:10.1007/s12020-026-04727-z)
Supplement: Supplementary file 2 [file 12020_2026_4727_MOESM2_ESM.docx]

**Table 1.** Characteristics of included studies

| **Author; Country; Publication Year** | **Study Design** | **Timing Definition** | **Timing of Early Protocol** | **Sample Size (n)**  **Early/Delayed** | **Age (mean)**  **Early/Delayed** | **Gender**  **(% Females)**  **Early/Delayed** | **RAI Dose**  **Early/Delayed**  **(GBq)** | **Follow-up Duration/ Assessment** | **Stimulation Agent** |
| --- | --- | --- | --- | --- | --- | --- | --- | --- | --- |
| Reza et al.;  Bangladesh; 2018 | Prospective quasi-experimental | Early: < 1h  Delayed: > 24h | 1 hour after RAI | 64/64 | NA^a^ | NA | NA | Up to 6 months; assessments at day 5, 3 months, and 6 months | Vitamin C; 1500 mg/day (250 mg every 3 h during daytime) for 7 days |
| Nakada et al.; Japan; 2005 | Prospective cohort | Early: < 1h  Delayed: > 24h | Within 1 hour of RAI | 105/125 | 55.2/58.5 | 85.7/88.8 | 3.96/3.87 | Up to 24 months; assessments during hospitalization and at regular follow-up intervals (1–6 months) | Lemon candy; 1–2 candies every 2–3 h during daytime for 5 days |
| Liu et al.; China; 2022 | Retrospective cohort | Early: < 2h  Delayed: > 24h | 2 hours after RAI | 230/230 | 45.06/45.29 | 71.3/70.8 | 4.07/3.70^b^ | Up to 6 months; clinical follow-up and salivary gland scintigraphy at 4–6 months | Vitamin C; 100 mg hourly during waking hours for 5 days |

h: hours; NA: not available

^a^The study reported only an overall age range of 21–65 years.

^b^RAI activities originally reported in millicuries (mCi) and were converted to gigabecquerels (GBq) using 1 mCi = 0.037 GBq.
